# Exploring Motives for Reducing Alcohol Consumption Among Users of an Alcohol Reduction App: Content Analysis

**DOI:** 10.2196/88992

**Published:** 2026-04-29

**Authors:** Lucille P Seppi, Olivia M Maynard, Dimitra Kale, John A Cunningham, Claire Garnett

**Affiliations:** 1 School of Psychological Science, University of Bristol, 12a Priory Road, Bristol, BS8 1TU, United Kingdom, 44 117 456 0728; 2 Department of Behavioural Science and Health, University College London, London, United Kingdom; 3 Department of Addictions Sciences, King's College London, London, United Kingdom; 4 Centre for Addiction and Mental Health, University of Toronto, Toronto, ON, Canada

**Keywords:** alcohol, motives to reduce, drinkers, United Kingdom, content analysis

## Abstract

**Background:**

It is important to understand the motives behind why people want to reduce their alcohol consumption to inform messaging for public health campaigns.

**Objective:**

This study aimed to identify the motives for reducing alcohol consumption among users of the Drink Less app in the United Kingdom.

**Methods:**

Content analysis of Drink Less app users’ responses to the prompt “I want to drink less because...” Users were aged 18 years or older, lived in the United Kingdom, and had downloaded the app between May 20, 2016, and June 24, 2024 (n=2520). Inductive content analysis was conducted to analyze users’ motives to drink less, and the frequencies were stratified by age, sex, and Alcohol Use Disorders Identification Test (AUDIT) score categories.

**Results:**

The most common motives to drink less were wanting to improve their physical health (1329/2520, 52.7%), feel better in their body (823/2520, 32.7%), improve their mental well-being (567/2520, 22.5%), regain agency (483/2520, 19.2%), live a different life (321/2520, 12.7%), and have better relationships (309/2520, 12.3%). The motives for drinking less,“improve physical health” and “feel better in their body,” had a lower prevalence among users in higher AUDIT risk zones compared with low-risk, while wanting to “improve their mental well-being,” “regain agency,” “live a different life,” and “have better relationships” had a higher prevalence among users in higher AUDIT risk zones.

**Conclusions:**

Users of an alcohol reduction app in the United Kingdom most commonly reported wanting to improve their physical health, feel better in their bodies, and improve their mental well-being as their motives for drinking less alcohol. The pattern of prevalence of different motives varied by AUDIT risk zones, highlighting the importance of tailoring interventions based on what is most likely to be motivating for individuals.

## Introduction

Alcohol consumption is a leading risk factor for early mortality, ill health, and disability in the United Kingdom [1], as well as contributing to health inequalities [2]. There is a dose-response relationship between the volume of alcohol consumed and the risk of alcohol-related harm (eg, stroke) [3], meaning anyone who drinks alcohol can benefit from reducing their consumption. It is important to understand the motives behind why people want to reduce their alcohol consumption to inform messaging for public health campaigns and create targeted guidance based on high-risk groups.

As of December 2025, around a third of risky drinkers had made an attempt in the past year to cut down or stop drinking, and a third believed they should or wanted to cut down [4]. Motivation is an important part of behavior change [5], and there is evidence that high motivation levels predict whether someone makes an attempt to reduce their consumption [6]. Motivation is commonly addressed in guidance documents and treatment manuals for brief alcohol interventions, and in the Cochrane Review of brief alcohol interventions in the form of identifying reasons for wanting to reduce alcohol consumption [7]. Having personally relevant motives may help to increase the level of motivation and lead more people to make a reduction attempt. Therefore, it is important to understand, among those who are motivated to make a reduction attempt, what the motive behind this was. This could be used to inform public health campaigns, with common motives highlighted to encourage others to make a reduction attempt as well.

Previous research has looked at understanding people’s motives for reducing their alcohol consumption. Adults in England reported motives to improve fitness, weight loss, future health concerns, social factors, cost, advice from a health care professional, and fewer social occasions [8-10]. Adults in Australia reported that health, lifestyle, and social motives were most common [11], alongside fear of the negative consequences among younger age groups [12,13]. People drinking at heavier levels reported motives such as major life changes, weighing up pros and cons of drinking and not drinking, health and financial concerns, and being affected by seeing someone drunk or high [14,15].

However, there are some gaps in the previous research. Some studies only included risky drinkers, so no one who had reduced their drinking to low-risk levels was asked about their motives [8,9]. In the cases of population surveys [8-11], very heavy drinkers tend to be underrepresented [16] despite being at greater risk of alcohol-related harm. In most previous research, participants were asked to select their motives for drinking less from a list of response options [8-12,14], and the process of selecting or deriving the response options was unclear. And finally, the timing of the data collection in these studies was either retrospective, introducing scope for recall bias [8-11], or participants were asked to report why they were considering changing their drinking behavior [15].

The Drink Less app is a theory-based intervention to support users in reducing their alcohol consumption in the United Kingdom that has been developed and refined in a systematic way involving user feedback [17,18] with evidence for its effectiveness [19]. It is widely used [20] and popular among its users, with a 4.6-star rating on the UK Apple App Store as of February 2026 [21]. Drink Less is one of the top apps for alcohol-related searches and has over 90,000 unique users [21]. The app collects anonymous data on users’ motives for drinking less in the form of an open-text response to the prompt “I want to drink less because...” These data can provide insight on motives to reduce alcohol consumption among UK drinkers at the moment at which they are making such an attempt. The aim of this study was to identify the Drink Less users’ motives for reducing their alcohol consumption.

## Methods

### Study Design

Qualitative analysis of previously collected short-text responses to the prompt “I want to drink less because...” among users of the Drink Less app. The study protocol was preregistered on the Open Science Framework (osf.io/gnb8p) prior to data analysis starting.

### Participants

The Drink Less app is freely available on the UK Apple App Store [21]. App users had to agree to the app’s “Privacy Policy” and “Terms and Conditions” for their anonymous data to be used for scientific research purposes. In addition to this, for this study, users had to be aged 18 years or older, live in the United Kingdom, and have downloaded the Drink Less app between May 18, 2016, and July 20, 2024 (there was no minimum engagement required) to be included in this study. In cases of duplicate device IDs, only the first download was included.

### The Drink Less App

The Drink Less app consists of 8 evidence-based modules to help users change their drinking behavior: Goal Setting, Self-Monitoring and Feedback, Action Planning, Normative Feedback, Cognitive Bias Re-Training, Insights, Behavioral Substitution, and Information About Antecedents [17,18]. The app was designed to help people reduce their alcohol consumption though there was no explicit user motive for reducing alcohol consumption that it was designed for or targeted on. Users could access all parts of the app at all times. The onboarding process (when first downloading the app) involves users agreeing to the app’s “Privacy Policy” and “Terms and Conditions” (they can opt out at any time, and if they do not agree, they can still use the app but not have their data used for scientific research purposes). During the time period of this study, research studies were conducted within the app (eg, [19,22]) though none of these affected the functionality or visibility of the “I want to drink less because...” prompt.

### Measures

#### Motive to Drink Less

All users received the prompt “I want to drink less because...” on the app dashboard and when setting goals though did not have to complete it. There was an open-text box for users to respond. If users changed their original response, the most recent version was used. There was no time limit on when the response had to be made by.

#### Demographic Characteristics

During app onboarding, users input their sex (male/female) and their year of birth (which was used to calculate their age). There were no other additional sociodemographic characteristics measured consistently across the time period to minimize participant burden and increase engagement with the app.

#### Alcohol Use Disorders Identification Test (AUDIT) Score

Users completed the AUDIT, a 10-item questionnaire with total scores ranging from 0 to 40 categorized into 4 risk zones [23]: zone I (score 0‐7, low-risk); zone II (score 8‐15, increasing risk); zone III (score 16‐19, higher risk); and zone IV (score 20‐40, at risk of alcohol dependence). This was completed during onboarding (ie, at the time of app download).

### Data Analysis

An inductive content analysis was conducted to analyze the users’ motives to drink less, allowing for large amounts of textual data to be explored qualitatively, identifying categories which can be quantified in terms of frequency [24]. This allows for patterns and meanings to be reported in a simpler manner with the aim of representing new insights and knowledge practically, guiding future action [25].

Researcher LPS familiarized themselves with the data and coded it inductively in Microsoft Excel using an iterative process that led to the development of *subthemes*, which came together to form *themes* and ultimately into *categories*. The criteria for selection were broad in that everything mentioned was considered as a motivation and not discounted for not fitting the previous literature, considering that our aim was to inductively explore motivations to drink less. Furthermore, the analysis was limited to a descriptive level so as to not become too abstract or look for underlying meaning due to both the volume of data and the short-form text (which cannot provide context or insight into potential underlying meanings in the way long-form text or interviews can) [26]. Each user response could be coded multiple times. Once an initial codebook came together, LPS met with CG and OMM to discuss it, for example, whether anything had been overlooked or something may have more than one meaning, after which LPS returned to the coding individually, shaped by the discussion. As all available data on motives to drink less were coded, inductive thematic saturation (when new codes or themes are rarely or never generated) [27] was not considered as a marker at which to stop coding, though it was reached before the end of the coding process. Each response was coded by its content, up until the last response. The prevalence of users reporting each subtheme, theme, and category was quantified.

The frequency of categories and themes was also stratified by age group, sex, and AUDIT risk zones. There was a change to the preregistered protocol in that subthemes were not stratified by age group, sex, and AUDIT risk zones due to very small cell sizes.

### Ethical Considerations

Ethics approval for the use of the Drink Less data for research purposes has been granted by University College London (CEHP/2020/579) and the University of Bristol (#23313) ethics committees. This particular study was approved by the School of Psychological Science Research Ethics Committee at the University of Bristol (#22068). The app has a privacy notice, and users provided consent for their anonymized data to be used for academic research purposes after downloading the app. Users can opt out from their data being used for academic research purposes within the app at any point. Participants were not compensated for taking part in this study.

## Results

### Overview

There were 93,640 unique users of the Drink Less app between May 20, 2016, and June 24, 2024, and 2550 (2.7%) of these users had responded to the prompt “I want to drink less because...” Of these, 30 responses were excluded due to being uninterpretable (eg, incoherent, not in English) (n=26), the user being under 18 years (n=3), or having missing demographic data (n=1).

The final sample consisted of 2520 unique user responses. The majority of users were between the ages of 35 and 54 years (n=1488, 59.1%) and identified as female (n=1594, 63.3%). Fewer than 5% (n=123) of users were categorized as a low-risk drinker, 57.2% (n=1441) as an increasing or higher risk drinker, and 37.9% (n=956) as at risk of alcohol dependence (see Table 1). The sample (ie, users completing the prompt) had a higher proportion of users who were younger (less than 54 years old), a higher proportion of female users, and a higher proportion of users at higher risk or risk of alcohol dependence compared with those not completing the prompt (see Table 1).

**Table 1.** Characteristics of users of the Drink Less app (2016-2024), including all users (n=93,640) and sample users (n=2520).

Characteristics	All users (n=93,640)	Users not completing the prompt (n=91,120)	Sample users (n=2520)	Chi-square (*df*)	*P* value
Age (years), n (%)				51.1 (5)	<.001
18‐24	4124 (4.4)	4038 (4.4)	86 (3.4)		
25‐34	15,045 (16.1)	14,614 (16.0)	431 (17.1)		
35‐44	24,143 (25.8)	23,405 (25.7)	738 (29.3)		
45‐54	27,295 (29.1)	26,545 (29.1)	750 (29.8)		
55‐64	17,170 (18.3)	16,748 (18.4)	422 (16.7)		
65+	5863 (6.3)	5770 (6.3)	93 (3.7)		
Sex, n (%)				202.6 (1)	<.001
Male	47,522 (50.7)	46,596 (51.1)	926 (36.7)		
Female	46,118 (49.3)	44,524 (48.9)	1594 (63.3)		
AUDIT^a^ risk zone, n (%)				290.4 (3)	<.001
I (score 0-7; low risk)	10,679 (11.4)	10,556 (11.6)	123 (4.9)		
II (score 8-15; increasing risk)	41,614 (44.4)	40,701 (44.7)	913 (36.2)		
III (score 16-19; higher risk)	17,062 (18.2)	16,534 (18.1)	528 (21.0)		
IV (score 20-40; at risk of alcohol dependence)	24,285 (25.9)	23,329 (25.6)	956 (37.9)		

a AUDIT: Alcohol Use Disorders Identification Test.

Of the sample users, the time difference between downloading the app and the date of the last update to users’ drink less motive had a median value of 0.9 (IQR 0.6‐6.6), indicating that over half of users reported their drink less motive within a day and three-quarters had reported their motive within 7 (mean 16.3, SD 57.8) days.

There were 19 categories, 29 themes, and 96 subthemes. On average, each response was coded 2.1 times, resulting in 5362 codes. The frequency of each category and theme is reported in Table 2, and the frequency of each category stratified by age, sex, and AUDIT risk zone is reported in Table 3. The frequency of users reporting each category and theme, and stratified by age, sex, and AUDIT risk zone, is reported in Multimedia Appendix 1. The frequency of subthemes is reported in Multimedia Appendix 2.

**Table 2.** Frequency of categories and themes of motives to drink less reported by users of the Drink Less app (2016-2024; sample users: n=2520).

Categories and themes^a^	Sample users (n=2520), n (%)
I want to improve my physical health	1329 (52.7)
I want to live a healthier lifestyle	1129 (85.0)
I am worried about future health problems	180 (13.6)
I have other health problems	72 (5.4)
I want to feel better in my body	823 (32.7)
Help with weight loss	718 (87.2)
Improve my fitness	113 (13.7)
I want to improve how I look	48 (5.8)
I want to improve the features on my body	32 (3.9)
I want to feel more attractive/sexy	3 (0.4)
I want to improve my mental well-being	567 (22.5)
I want to improve my overall well-being	346 (61.0)
Improve mental health	198 (34.9)
I want to feel clearer-headed, more alert, focused/be present	87 (15.3)
I want to regain agency	483 (19.2)
I want to be in control of my drinking	330 (68.3)
I want to be in control (of my actions, life...)	114 (23.6)
I do not want drinking to define me	93 (19.3)
I want to live a different life	321 (12.7)
I want to make space in my life for other things	136 (42.4)
I want to live (longer)	91 (28.3)
I want to be more...	83 (25.9)
I want to improve my quality of life	46 (14.3)
To have better relationships with the people in my life	309 (12.3)
I am doing it for someone	261 (84.5)
Drinking affects my relationships/social life	48 (15.5)
My drinking affects the way I treat and impact people	21 (6.8)
I want to improve my sex life	4 (1.3)
A decision that drinking was too expensive	228 (9.1)
Restore my energy	212 (8.4)
I do not want to (re)experience the side effects/consequences	162 (6.4)
Drinking hurts me	94 (3.7)
I want to have a better relationship with alcohol	64 (2.5)
I want to moderate my drinking, not abstain	61 (95.3)
Temporary abstinence (eg, Dry January)	4 (6.3)
I have changed my attitude about drinking	43 (1.7)
To support or encourage people in my life to drink less/have a better relationship with alcohol	43 (1.7)
Improve work life	40 (1.6)
I am planning for my future (eg, starting a family/finding a partner/planning for retirement)	36 (1.4)
Future family planning	19 (52.8)
Planning for retirement/old age	17 (47.2)
Other	30 (1.2)
Someone or something inspired me to	28 (1.1)
Advice or concern	26 (92.3)
Media	2 (7.1)
I have seen the effects drinking has had on other people	17 (0.7)
I do not want to end up like someone I know	17 (100.0)
A significant holiday or event (eg, birthday or upcoming trip)	17 (0.7)

a The percentage of users for all categories and for themes within each category may be greater than 100% because each response could be coded multiple times.

**Table 3.** Frequency of categories of motives to drink less reported by users of the Drink Less app, and stratified by sociodemographic (age and sex) and drinking characteristics (AUDIT^a^ risk zone) (sample users: n=2520)^b^.

		Age in years, n (%)						Sex, n (%)		AUDIT risk zone^c^, n (%)			
Category	All users (n=2520), n (%)	18‐24 (n=86)	25‐34 (n=431)	35‐44 (n=738)	45‐54 (n=750)	55‐64 (n=422)	65+ (n=93)	Male (n=926)	Female (n=1594)	I (n=123)	II (n=913)	III (n=528)	IV (n=956)
I want to improve my physical health	1329 (52.7)	30 (34.9)	194 (45.0)	398 (53.9)	391 (52.1)	257 (60.9)	59 (63.4)	480 (51.8)	849 (53.3)	76 (61.8)	505 (55.3)	291 (55.1)	457 (47.8)
I want to feel better in my body	823 (32.7)	19 (22.1)	116 (26.9)	247 (33.5)	271 (36.1)	151 (35.8)	19 (20.4)	251 (27.1)	572 (35.9)	48 (39.0)	322 (35.3)	182 (34.5)	271 (28.3)
I want to improve my mental well-being	567 (22.5)	23 (26.0)	124 (28.8)	181 (24.5)	177 (23.6)	49 (11.6)	13 (14.0)	171 (18.5)	396 (24.8)	13 (10.6)	185 (20.3)	126 (23.9)	243 (25.4)
I want to regain agency	483 (19.2)	24 (27.9)	93 (21.6)	130 (17.6)	139 (18.5)	80 (19.0)	17 (18.3)	146 (15.8)	337 (21.1)	11 (8.9)	142 (15.6)	98 (18.6)	232 (24.3)
I want to live a different life	321 (12.7)	8 (9.3)	67 (15.5)	117 (15.9)	83 (11.1)	39 (9.2)	7 (7.5)	119 (12.9)	202 (12.7)	6 (4.9)	94 (10.3)	69 (13.1)	152 (15.9)
To have better relationships with the people in my life	309 (12.3)	8 (9.3)	60 (13.9)	133 (18.0)	81 (10.8)	21 (5.0)	6 (6.4)	136 (14.7)	173 (10.9)	6 (4.9)	59 (6.5)	65 (12.3)	179 (18.7)
A decision that drinking was too expensive	228 (9.1)	8 (9.3)	44 (10.2)	80 (10.8)	61 (8.1)	32 (7.6)	3 (3.2)	90 (9.7)	138 (8.7)	8 (6.5)	68 (7.4)	58 (11.0)	94 (9.8)
Restore my energy	212 (8.4)	5 (5.8)	32 (7.4)	61 (8.3)	77 (10.3)	30 (7.1)	7 (7.5)	51 (5.5)	161 (10.1)	9 (7.3)	90 (9.9)	47 (8.9)	66 (6.9)
I do not want to (re)experience the side effects/consequences	162 (6.4)	7 (8.1)	30 (7.0)	52 (7.0)	45 (6.0)	21 (5.0)	7 (7.5)	35 (3.8)	127 (8.0)	5 (4.1)	42 (4.6)	43 (8.1)	72 (7.5)
Drinking hurts me	94 (3.7)	5 (5.8)	16 (3.7)	28 (3.8)	25 (3.3)	12 (2.8)	8 (8.6)	36 (3.9)	58 (3.6)	3 (2.4)	21 (2.3)	20 (3.8)	50 (5.2)
I want to have a better relationship with alcohol	64 (2.5)	3 (3.5)	9 (2.1)	21 (2.8)	17 (2.3)	11 (2.6)	3 (3.2)	20 (2.2)	44 (2.8)	4 (3.3)	23 (2.5)	13 (2.5)	24 (2.5)
I have changed my attitude about drinking	43 (1.7)	2 (2.3)	4 (0.9)	15 (2.0)	18 (2.4)	1 (0.2)	3 (3.2)	19 (2.1)	24 (1.5)	3 (2.4)	14 (1.5)	11 (2.1)	15 (1.6)
To support or encourage people in my life to drink less/have a better relationship with alcohol	43 (1.7)	0 (0.0)	5 (1.2)	20 (2.7)	17 (2.3)	1 (0.2)	0 (0.0)	13 (1.4)	30 (1.9)	0 (0.0)	18 (2.0)	13 (2.5)	12 (1.3)
Improve work life	40 (1.6)	3 (3.5)	12 (2.8)	15 (2.0)	9 (1.2)	1 (0.2)	0 (0.0)	17 (1.8)	23 (1.4)	1 (0.8)	8 (0.9)	12 (2.3)	19 (2.0)
I am planning for my future (eg, starting a family/finding a partner/planning for retirement)	36 (1.4)	1 (1.2)	7 (1.6)	14 (1.9)	8 (1.1)	6 (1.4)	0 (0.0)	13 (1.4)	23 (1.4)	1 (0.8)	16 (1.8)	7 (1.3)	12 (1.3)
Other	30 (1.2)	1 (1.2)	6 (1.4)	5 (0.7)	11 (1.5)	6 (1.4)	1 (1.1)	17 (1.8)	13 (0.8)	1 (0.8)	15 (1.6)	8 (1.5)	6 (0.6)
Someone or something inspired me to	28 (1.1)	2 (2.3)	6 (1.4)	6 (0.8)	11 (1.5)	2 (0.5)	1 (1.1)	12 (1.3)	16 (1.0)	3 (2.4)	7 (0.8)	5 (0.9)	13 (1.4)
I have seen the effects drinking has had on other people	17 (0.7)	2 (2.3)	3 (0.7)	2 (0.3)	9 (1.2)	1 (0.2)	0 (0.0)	4 (0.4)	13 (0.8)	1 (0.8)	3 (0.3)	4 (0.8)	9 (0.9)
A significant holiday or event (eg, birthday or upcoming trip)	17 (0.7)	0 (0.0)	8 (1.9)	3 (0.4)	5 (0.7)	1 (0.2)	0 (0.0)	5 (0.5)	12 (0.8)	0 (0.0)	6 (0.7)	5 (0.9)	6 (0.6)

a AUDIT: Alcohol Use Disorders Identification Test.

b The percentage of users for all categories may be greater than 100% because each response could be coded multiple times.

c Zone I (score 0-7), zone II (score 8-15), zone III (score 16-19), and zone IV (score 20-40).

### Category 1: I Want to Improve My Physical Health

Over half of users reported wanting to improve physical health (1329/2520, 52.7%), and most responses were under the theme “wanting to live a healthier lifestyle” (n=1129, 85%; such as unspecified goals to take care of one’s general health or specific goals like lowering one’s cholesterol), in contrast with being “worried about future health problems” (n=180, 13.6%; specific concerns such as future liver failure or cancer). The least common theme was having other health problems, which directly inspired a reduction attempt, including motives to stop the exacerbation of health problems caused through or impacted on by alcohol. This theme also described motives where alcohol reduction was not the primary goal, such as reducing consumption to aid recovery from something else.

The “improving physical health” category was reported by users across all age groups, increasing in prevalence from 34.9% (30/86) among 18‐24 year olds to 63.4% (59/93) among those over 65 years old. This category had similar prevalence among male and female users. The prevalence of users reporting wanting to improve physical health was higher among those in AUDIT risk zone I (76/123, 61.8%), with prevalence at a lower level (457/956, 47.8%) among those in risk zone IV.

### Category 2: I Want to Feel Better in My Body

Nearly a third of users (823/2520, 32.7%) reported wanting to feel better in their body. The most common theme was “Help with weight loss” (718/823, 87.2%), followed by “Improve my fitness” (113/823, 13.7%), consisting of either a general motive to do so or wanting to improve in a specific sport or exercise. The other themes were “I want to improve how I look” (48/823, 5.8%), “I want to improve the features on my body” (32/823, 3.9%), mainly referring to the skin, and “I want to feel more attractive/sexy” (3/823, 0.4%).

All age groups reported wanting to feel better in their body (20.4% to 36.1%), with prevalence highest across age groups 35‐64 years old. More female users reported wanting to feel better in their body (572/1594, 35.9%) compared with male users (251/926, 27.1%). The prevalence of reporting wanting to feel better in their body was highest among AUDIT risk zone I (48/123, 39%) and lowest in prevalence among risk zone IV (271/956, 28.3%).

### Category 3: I Want to Improve My Mental Well-Being

Around a quarter of users reported wanting to improve their mental well-being (567/2520, 22.5%), with the most common theme being to improve their overall well-being (346/567, 61%), mainly wanting to feel better or not wanting drinking to impact on their mood. Some also specified wanting a better relationship with themselves as well as to feel less irritable, stressed, or frustrated. The other common theme was improving mental health (198/567, 34.9%), which specified motives to reduce the symptoms of mental health conditions such as anxiety and depression, and a general desire to improve mental health.

It was more common to report wanting to improve mental well-being as a motive to drink less among users in the age groups of 18‐54 years (over 23.6%). The prevalence of reporting wanting to improve mental well-being was slightly higher among female users (396/1594, 24.8%) compared with male users (171/926, 18.5%). Reporting wanting to improve mental well-being was highest among users in AUDIT risk zone IV (243/956, 25.4%) and lowest in prevalence among risk zone I (13/123, 10.6%).

### Category 4: I Want to Regain Agency

Wanting to regain agency was reported by 19.2% (483/2520) of users, with the most common theme being “I want to be in control of my drinking” (330/483, 68.3%), which mainly consisted of a fear of becoming or being dependent as well as feeling guilty or ashamed about one’s drinking or expressing concern about usually drinking more than intended. A small percentage of users wanted to make themselves or others proud or prove that they could reduce their consumption, whereas others felt that they “have to” or “need to” reduce for unspecified reasons.

Within this category, a quarter of users (114/483, 23.6%) expressed wanting to be in control over more than just their drinking, such as their actions or life. This consisted of users reporting wanting to be more responsible/reliable and the feeling that alcohol prevents them from making “good” choices. Lastly, some users mentioned that they did not want drinking to define them (93/483, 19.3%), which mainly referred to issues of identity (ie, feeling more like oneself without drink), but also not wanting their drinking to embarrass them or others, and not wanting others to worry about them.

Over a quarter of users aged 18‐24 years reported this category of wanting to regain agency (24/86, 27.9%) compared with slightly lower prevalence (17.6% to 21.6%) among other age groups. More female users (337/1594, 21.1%) reported wanting to regain agency as a motive to drink less compared with 15.8% (146/926) of male users. Reporting wanting to regain agency was highest among users in AUDIT risk zone IV (232/956, 24.3%) and lowest in risk zone I (11/123, 8.9%).

### Category 5: I Want to Live a Different Life

Wanting to live a different life was reported by 12.7% (321/2520) of users. The most common theme was “I want to make space in my life for other things” (136/321, 42.4%), then “I want to live (longer)” (91/321, 28.3%) and “I want to be more” (83/321, 25.9%) of a certain quality, such as productive, successful, or resilient.

This category was more prevalent among users aged 25‐44 years (~15%) compared with other age groups and was similarly prevalent between male (119/926, 12.9%) and female users (202/1594, 12.7%). The prevalence of wanting to live a different life was highest among users in the AUDIT risk zone IV (152/956, 15.9%) and lowest among those users in risk zone I (6/123, 4.9%).

### Category 6: To Have Better Relationships With the People in My Life

Wanting to have better relationships with the people in their life as a motive to drink less was reported by 12.3% (309/2520) of users. The main theme of this category was “I am doing it for someone” (261/309, 84.5%), such as family, partners, friends, or someone unspecified. Other themes included “Drinking affects my relationships/social life” (48/309, 15.5%) and “My drinking affects the way I treat and impact people” (21/309, 6.8%).

The prevalence of wanting to have better relationships with the people in their life was higher among the 25‐34 and 35‐44 years age groups (60/431 [13.9%] and 133/738 [18%], respectively) and lower among other age groups. A higher proportion of male users reported this motive to drink less (136/926, 14.7%) compared with female users (173/1594, 10.9%). The prevalence of wanting to have better relationships with the people in their life was highest among users in AUDIT risk zone IV (179/956, 18.7%) and lowest among users in risk zone I (6/123, 4.9%).

### Other Categories

All other categories for motives to drink less were mentioned by fewer than 10% of users: deciding that drinking was too expensive (228/2520, 9.1%), restoring energy (212/2520, 8.4%), not wanting to experience the side effects/consequences (162/2520, 6.4%), drinking hurts me (94/2520, 3.7%), wanting to have a better relationship with alcohol (64/2520, 2.5%), changed attitude about drinking (43/2520, 1.7%), supporting others to drink less (43/2520, 1.7%), improve work life (40/2520, 1.6%), planning for future (36/2520, 1.4%), other (30/2520, 1.2%), being inspired to (28/2520, 1.1%), seen the effects drinking has on others (17/2520, 0.7%), and a significant event (17/2520, 0.7%).

## Discussion

### Main Findings

The most common motives to reduce drinking among users of the Drink Less app in the United Kingdom were wanting to improve their physical health, feel better in their body, improve their mental well-being, and regain agency. These participants, by the nature of the sample, had all taken some action to download and start using an evidence-based digital intervention with evidence for its effectiveness [19], suggesting that these individuals were in either the “action” stage of change or perhaps in “preparation” [28], having downloaded the app but may not have planned on using it immediately. Wanting to improve their physical health was reported as a motive to drink less by over half of users, and this was present across all age groups though increased with age. Nearly a third of users reported wanting to feel better in their body (eg, weight loss and improved fitness), and this was more common among female users. Interestingly, there were clear patterns for the prevalence of motives across users’ AUDIT risk zones. “Improving physical health” and “wanting to feel better in their body” were highest among users drinking at low-risk levels and decreased in prevalence with higher AUDIT risk zones (though in absolute terms, these motives were still higher than for other motives for drinking less).

In contrast, motives to “improve their mental well-being,” “regain agency,” “live a different life,” and “have better relationships” all had the highest prevalence among users at risk of alcohol dependence and the lowest among those drinking at low risk. About a quarter of users reported wanting to improve their mental well-being, and this motive was reported more among users aged between 18 and 54 years and was slightly more common among female users. Around a fifth of users reported wanting to regain agency, mainly reporting wanting to be in control of their drinking and being worried about dependency. About a tenth reported wanting to live a different life and to have better relationships with the people in their life.

### Comparison With Other Studies

The findings of this study that physical health and wanting to feel better in their body were common motives for drinking less is consistent with previous research [8-11,14,15,29], including that these motives were more commonly reported by older age groups. This study found that improving mental health was a relatively common motive to drink less though this motive is not typically seen across previous research, where there tends to be a focus on physical health, or health more generally. There were also some motives that came up in previous research, such as fewer social occasions [10] and treatment or therapy [15], that were not common in this study. This could be due to differences in the samples with participants in the current study being users of an alcohol reduction app and therefore consciously trying to reduce their alcohol consumption. The motive to drink less due to worries around dependency tended not to be included as a response option in previous research but came up in this study and another study where participants could give free responses [15]. The samples in these two studies both included heavier drinkers, highlighting the importance of sampling heavier drinkers when assessing motives for drinking less as an important group to consider for tailoring public health interventions.

This study found motives to drink less that were about seeking a positive outcome (eg, wanting to feel happier and have better relationships with themselves) and not just avoiding a negative outcome (eg, not wanting to experience the consequences and worries about dependency). Another study asking participants about their motives to drink less without using preset response options found similarly positive reasons for reducing alcohol consumption [15].

A previous study found differences in motives based on the level of severity of alcohol use with “consequence-related” reasons (such as health problems and financial concerns) more common among those with more severe alcohol use, and “drifting out” reasons (changes in circumstances such as having children) more common among those with less severe alcohol use [30]. While the current study saw different patterns in motives to drink less based on AUDIT risk zone, it did not follow the same pattern as the previous study with physical health and feeling better in their body (both “consequence-related” reasons) being more common among app users with low-risk AUDIT zones.

### Strengths and Limitations

A major strength of this study was that data collection was remote and in a naturalistic setting, meaning these findings are likely to have high external validity. Participants were also those that were currently trying to reduce their alcohol consumption and prompted to enter their motive (as free text) for drinking less when they downloaded the app, avoiding issues around recall bias. The app is also a stand-alone intervention, so no researcher was present, avoiding potential fear of judgment or social conformity [31]. Data were also collected from users with a range of ages and AUDIT scores, therefore, capturing data from heavier drinkers and people at risk of alcohol dependence, which is sometimes underrepresented in household surveys [16].

This study collected data from 2016 to 2024, though we did not adjust for time periods such as the COVID-19 pandemic, which are known to have affected the prevalence of attempts to reduce alcohol consumption in the United Kingdom [32]. Previous research has looked at time trends in motives for attempts to reduce alcohol consumption from a nationally representative cross-sectional survey and saw increases in attempts motivated by health concerns and those motivated by cost and social factors [9]. Future research could look to identify any trends in the prevalence of different alcohol reduction motives among this user group over time to see if this reflects what was seen across the general population.

The app users included in this study, who reported their motive for drinking less in the app, were only a small proportion of total app users (fewer than 3%) and differed by their age, sex, and AUDIT risk zone. Therefore, this sample is self-selected and not generalizable to all users of the Drink Less app. However, not completing the prompt is unlikely to have a bearing on users’ readiness to change given the level of motivation already likely present; participants had downloaded and completed the onboarding process for an app named “Drink Less,” which has a description in the UK App Store of “Are you looking to cut down how much you drink? If so, we can help.” Also, there is previous evidence that the extent of behavioral engagement (eg, time spent on the app) with Drink Less does not have a mediating effect on its effectiveness, suggesting that these users who have greater behavioral engagement with the app and report their motive for drinking less are not more or less likely to be successful in reducing their alcohol consumption [33]. Therefore, we believe these findings provide useful information on what actually motivates an attempt to reduce alcohol consumption even though they are from a self-selected sample of users, and these may generalize to other people motivated to reduce their alcohol consumption but not using the app. Further to this, we only analyzed data on motives to drink less alcohol from one app. While the Drink Less app is a widely used, popular [21] and evidence-based app [19], future research could explore whether similar motives to drink less are reported in other alcohol reduction apps.

The vast majority of motives to drink less were reported within 1 week of downloading the app suggesting that these motives related to why they had made an attempt to reduce their drinking (by downloading the Drink Less app). While we do not know if users sustained their motive throughout their attempt, they were able to update it at any point. A quarter of users last updated their motive to drink less over a week after downloading the app, meaning that their AUDIT score (recorded on the day of app download) may have changed since onboarding. However, while the AUDIT-C (the first 3 items) is responsive to change [34], the full 10-item AUDIT is likely to be less responsive to change given the nature of the remaining 7 items. Further to this, in this study, we categorized users by their AUDIT risk zone (ie, scores ranging from 0 to 40 grouped into 4 categories), meaning a greater change in AUDIT score would be required for the user to change AUDIT risk zone.

Another limitation of this study was that the content analysis was conducted manually by one researcher, with no interrater reliability calculated.

### Future Research

Understanding people’s motivations to reduce their alcohol consumption has important implications for how to motivate risky drinkers to make reduction attempts given that identifying these motives is commonly addressed across guidance documents and treatment manuals for brief alcohol interventions [7]. We found different patterns in motives to drink less for people at either end of the AUDIT risk zone, which could be further explored in other populations and potentially used to inform tailoring of public health campaigns and interventions. This study’s findings can be used to inform materials or public health campaigns encouraging risky drinkers to use the Drink Less app with examples of other users’ motives, rather than to only consume less alcohol given the skepticism and denial of national alcohol guidelines that some people have (eg, [35]).

By the nature of the sample, all the study participants were using an evidence-based digital intervention with evidence for its effectiveness for helping users reduce 2 UK units a week over a 6-month period [19], and therefore, their motives to drink less had all resulted in action to do something. However, we do not know whether there were certain types of motives which are more successful in reducing alcohol consumption than others, and this is something that we will look to investigate in the future.

The findings of this study also informed the response options for the question on motives for cutting down on alcohol consumption included in the Alcohol Toolkit Study [36] (with 2 new response options “Improve my mental health or well-being” and “To avoid bad experiences when drinking (aggressive behavior, hangovers, risk of injury, etc)” included).

### Conclusions

Users of an alcohol reduction app, Drink Less, in the United Kingdom most commonly reported improving their physical health, feeling better in their body, improving their mental well-being, regaining agency, living a different life, and having better relationships with the people in their life as their motives for reducing their alcohol consumption. Wanting to improve mental well-being, regain agency, live a different life, and to have better relationships were all motives that had the highest prevalence among people at risk of alcohol dependence and the lowest among those drinking at low-risk levels. Understanding the motives behind why people are attempting to reduce their alcohol consumption, particularly among heavier drinkers, could inform how to encourage more people to make an attempt to reduce their drinking.

## Supplementary material

10.2196/88992 Multimedia Appendix 1 Frequency of categories and themes of motives to drink less reported by app users, stratified by sociodemographic and drinking characteristics (n=2520).

10.2196/88992 Multimedia Appendix 2 Frequency of categories, themes, and subthemes reported by the Drink Less app users (n=2520).

## Abbreviations

AUDIT: Alcohol Use Disorders Identification Test
