# Supplementary material for: Exploring Motives for Reducing Alcohol Consumption Among Users of an Alcohol Reduction App: Content Analysis
Source: JMIR Public Health Surveill. 2026 Apr 29;12:e88992. doi: 10.2196/88992 (PMC13128053; doi:10.2196/88992)
Supplement: Multimedia Appendix 1 [file publichealth-v12-e88992-s001.docx]

**Frequency of categories and themes of motives to drink less reported by app users, and stratified by sociodemographic and drinking characteristics (n=2520)**

| **Category**  Theme | % (n) all users (n=2,520) | % Age in years (n) | | | | | | % Sex (n) | | % AUDIT risk zone (n) | | | |
| --- | --- | --- | --- | --- | --- | --- | --- | --- | --- | --- | --- | --- | --- |
|  |  | 18-24 (n=86) | 25-34 (n=431) | 35-44 (n=738) | 45-54 (n=750) | 55-64 (n=422) | 65+ (n=93) | Male (n=926) | Female (n=1,594) | I (n=123) | II (n=913) | III (n=528) | IV (n=956) |
| **I want to improve my physical health** | 52.7 (1329) | 34.9 (30) | 45.0 (194) | 53.9 (398) | 52.1 (391) | 60.9 (257) | 63.4 (59) | 51.8 (480) | 53.3 (849) | 61.8 (76) | 55.3 (505) | 55.1 (291) | 47.8 (457) |
| I want to live a healthier lifestyle | 85.0 (1129) | 83.3 (25) | 83.0 (161) | 87.2 (347) | 83.9 (328) | 84.0 (216) | 88.1 (52) | 85.4 (410) | 84.7 (719) | 86.8 (66) | 87.7 (443) | 81.8 (238) | 83.6 (382) |
| I am worried about future health problems | 13.6 (180) | 6.7 (2) | 13.4 (26) | 12.3 (49) | 15.1 (59) | 14.0 (36) | 13.6 (8) | 11.7 (56) | 14.6 (124) | 10.5 (8) | 12.5 (63) | 15.1 (44) | 14.2 (65) |
| I have other health problems | 5.4 (72) | 10.0 (3) | 6.7 (13) | 4.8 (19) | 4.6 (18) | 5.4 (14) | 8.5 (5) | 6.9 (33) | 4.6 (39) | 10.5 (8) | 3.4 (17) | 6.5 (19) | 6.1 (28) |
| **I want to feel better in my body** | 32.7 (823) | 22.1 (19) | 26.9 (116) | 33.5 (247) | 36.1 (271) | 35.8 (151) | 20.4 (19) | 27.1 (251) | 35.9 (572) | 39.0 (48) | 35.3 (322) | 34.5 (182) | 28.3 (271) |
| Help with weight loss | 87.2 (718) | 78.9 (15) | 85.3 (99) | 85.0 (210) | 87.5 (237) | 93.4 (141) | 84.2 (16) | 86.1 (216) | 87.8 (502) | 89.6 (43) | 88.5 (285) | 87.4 (159) | 85.2 (231) |
| Improve my fitness | 13.7 (113) | 10.5 (2) | 15.5 (18) | 16.6 (41) | 11.1 (30) | 11.9 (18) | 21.1 (4) | 20.3 (51) | 10.8 (62) | 16.7 (8) | 11.8 (38) | 14.3 (26) | 15.1 (41) |
| I want to improve how I look | 5.8 (48) | 5.3 (1) | 6.9 (8) | 6.5 (16) | 6.6 (18) | 2.6 (4) | 5.3 (1) | 3.6 (9) | 6.8 (39) | 0.0 (0) | 5.3 (17) | 6.0 (11) | 7.4 (20) |
| I want to improve the features on my body | 3.9 (32) | 5.3 (1) | 2.6 (3) | 6.5 (16) | 3.7 (10) | 1.3 (2) | 0.0 (0) | 1.6 (4) | 4.9 (28) | 4.2 (2) | 3.4 (11) | 3.8 (7) | 4.4 (12) |
| I want to feel more attractive/sexy | 0.4 (3) | 0.0 (0) | 0.0 (0) | 0.0 (0) | 0.7 (2) | 0.7 (1) | 0.0 (0) | 0.4 (1) | 0.3 (2) | 0.0 (0) | 0.0 (0) | 1.1 (2) | 0.4 (1) |
| **I want to improve my mental wellbeing** | 22.5 (567) | 26.0 (23) | 28.8 (124) | 24.5 (181) | 23.6 (177) | 11.6 (49) | 14.0 (13) | 18.5 (171) | 24.8 (396) | 10.6 (13) | 20.3 (185) | 23.9 (126) | 25.4 (243) |
| I want to improve my overall wellbeing | 61.0 (346) | 47.8 (11) | 55.6 (69) | 66.9 (121) | 57.1 (101) | 69.4 (34) | 76.9 (10) | 59.6 (102) | 61.6 (244) | 30.8 (4) | 62.2 (115) | 57.1 (72) | 63.8 (155) |
| Improve mental health | 34.9 (198) | 56.5 (13) | 46.8 (58) | 35.9 (65) | 26.0 (46) | 28.6 (14) | 15.4 (2) | 33.9 (58) | 35.4 (140) | 23.1 (3) | 30.8 (57) | 39.7 (50) | 36.2 (88) |
| I want to feel clearer headed, more alert, focused/be present | 15.3 (87) | 8.7 (2) | 7.3 (9) | 12.7 (23) | 26.6 (47) | 8.2 (4) | 15.4 (2) | 15.2 (26) | 15.4 (61) | 46.2 (6) | 20.0 (37) | 15.1 (19) | 10.3 (25) |
| **I want to regain agency** | 19.2 (483) | 27.9 (24) | 21.6 (93) | 17.6 (130) | 18.5 (139) | 19.0 (80) | 18.3 (17) | 15.8 (146) | 21.1 (337) | 8.9 (11) | 15.6 (142) | 18.6 (98) | 24.3 (232) |
| I want to be in control of my drinking | 68.3 (330) | 50.0 (12) | 58.1 (54) | 63.8 (83) | 74.8 (104) | 78.8 (63) | 82.4 (14) | 65.8 (96) | 69.4 (234) | 81.8 (9) | 73.2 (104) | 72.4 (71) | 62.9 (146) |
| I want to be in control (of my actions, life...) | 23.6 (114) | 25.0 (6) | 22.6 (21) | 26.9 (35) | 23.7 (33) | 20.0 (16) | 17.6 (3) | 24.0 (35) | 23.4 (79) | 9.1 (1) | 19.7 (28) | 21.4 (21) | 27.6 (64) |
| I do not want drinking to define me | 19.3 (93) | 33.3 (8) | 26.9 (25) | 23.1 (30) | 14.4 (20) | 10.0 (8) | 11.8 (2) | 17.8 (26) | 19.9 (67) | 18.2 (2) | 17.6 (25) | 17.3 (17) | 21.1 (49) |
| **I want to live a different life** | 12.7 (321) | 9.3 (8) | 15.5 (67) | 15.9 (117) | 11.1 (83) | 9.2 (39) | 7.5 (7) | 12.9 (119) | 12.7 (202) | 4.9 (6) | 10.3 (94) | 13.1 (69) | 15.9 (152) |
| I want to make space in my life for other things | 42.4 (136) | 50.0 (4) | 49.3 (33) | 41.9 (49) | 37.3 (31) | 43.6 (17) | 28.6 (2) | 37.8 (45) | 45.0 (91) | 66.7 (4) | 40.4 (38) | 49.3 (34) | 39.5 (60) |
| I want to live (longer) | 28.3 (91) | 0.0 (0) | 9.0 (6) | 34.2 (40) | 38.6 (32) | 25.6 (10) | 42.9 (3) | 28.6 (34) | 28.2 (57) | 0.0 (0) | 28.7 (27) | 26.1 (18) | 30.3 (46) |
| I want to be more ... | 25.9 (83) | 62.5 (5) | 31.3 (21) | 29.9 (35) | 18.1 (15) | 17.9 (7) | 0.0 (0) | 31.9 (38) | 22.3 (45) | 33.3 (2) | 26.6 (25) | 30.4 (21) | 23.0 (35) |
| I want to improve my quality of life | 14.3 (46) | 0.0 (0) | 16.4 (11) | 10.3 (12) | 15.7 (13) | 17.9 (7) | 42.9 (3) | 17.6 (21) | 12.4 (25) | 0.0 (0) | 12.8 (12) | 11.6 (8) | 17.1 (26) |
| **To have better relationships with the people in my life** | 12.3 (309) | 9.3 (8) | 13.9 (60) | 18.0 (133) | 10.8 (81) | 5.0 (21) | 6.4 (6) | 14.7 (136) | 10.9 (173) | 4.9 (6) | 6.5 (59) | 12.3 (65) | 18.7 (179) |
| I am doing it for someone | 84.5 (261) | 62.5 (5) | 70.0 (42) | 88.7 (118) | 92.6 (75) | 76.2 (16) | 83.3 (5) | 84.6 (115) | 84.4 (146) | 66.7 (4) | 96.6 (57) | 87.7 (57) | 79.9 (143) |
| Drinking affects my relationships/social life | 15.5 (48) | 37.5 (3) | 25.0 (15) | 13.5 (18) | 6.2 (5) | 23.8 (5) | 33.3 (2) | 16.2 (22) | 15.0 (26) | 16.7 (1) | 1.7 (1) | 10.8 (7) | 21.8 (39) |
| My drinking affects the way I treat and impact people | 6.8 (21) | 12.5 (1) | 11.7 (7) | 5.3 (7) | 4.9 (4) | 9.5 (2) | 0.0 (0) | 5.9 (8) | 7.5 (13) | 16.7 (1) | 3.4 (2) | 3.1 (2) | 8.9 (16) |
| I want to improve my sex life | 1.3 (4) | 0.0 (0) | 1.7 (1) | 2.3 (3) | 0.0 (0) | 0.0 (0) | 0.0 (0) | 0.0 (0) | 2.3 (4) | 0.0 (0) | 3.4 (2) | 1.5 (1) | 0.6 (1) |
| **A decision that drinking was too expensive** | 9.1 (228) | 9.3 (8) | 10.2 (44) | 10.8 (80) | 8.1 (61) | 7.6 (32) | 3.2 (3) | 9.7 (90) | 8.7 (138) | 6.5 (8) | 7.4 (68) | 11.0 (58) | 9.8 (94) |
| **Restore my energy** | 8.4 (212) | 5.8 (5) | 7.4 (32) | 8.3 (61) | 10.3 (77) | 7.1 (30) | 7.5 (7) | 5.5 (51) | 10.1 (161) | 7.3 (9) | 9.9 (90) | 8.9 (47) | 6.9 (66) |
| **I do not want to (re)experience the side effects/consequences** | 6.4 (162) | 8.1 (7) | 7.0 (30) | 7.0 (52) | 6.0 (45) | 5.0 (21) | 7.5 (7) | 3.8 (35) | 8.0 (127) | 4.1 (5) | 4.6 (42) | 8.1 (43) | 7.5 (72) |
| **Drinking hurts me** | 3.7 (94) | 5.8 (5) | 3.7 (16) | 3.8 (28) | 3.3 (25) | 2.8 (12) | 8.6 (8) | 3.9 (36) | 3.6 (58) | 2.4 (3) | 2.3 (21) | 3.8 (20) | 5.2 (50) |
| **I want to have a better relationship with alcohol** | 2.5 (64) | 3.5 (3) | 2.1 (9) | 2.8 (21) | 2.3 (17) | 2.6 (11) | 3.2 (3) | 2.2 (20) | 2.8 (44) | 3.3 (4) | 2.5 (23) | 2.5 (13) | 2.5 (24) |
| I want to moderate my drinking, not abstain | 95.3 (61) | 66.7 (2) | 100.0 (9) | 95.2 (20) | 100.0 (17) | 90.9 (10) | 100.0 (3) | 100.0 (20) | 93.2 (41) | 75.0 (3) | 95.7 (22) | 100.0 (13) | 95.8 (23) |
| Temporary abstinence (e.g. Dry January) | 6.3 (4) | 33.3 (1) | 11.1 (1) | 4.8 (1) | 0.0 (0) | 9.1 (1) | 0.0 (0) | 5.0 (1) | 6.8 (3) | 25.0 (1) | 8.7 (2) | 0.0 (0) | 4.2 (1) |
| **I have changed my attitude about drinking** | 1.7 (43) | 2.3 (2) | 0.9 (4) | 2.0 (15) | 2.4 (18) | 0.2 (1) | 3.2 (3) | 2.1 (19) | 1.5 (24) | 2.4 (3) | 1.5 (14) | 2.1 (11) | 1.6 (15) |
| **To support or encourage people in my life to drink less/have a better relationship with alcohol** | 1.7 (43) | 0.0 (0) | 1.2 (5) | 2.7 (20) | 2.3 (17) | 0.2 (1) | 0.0 (0) | 1.4 (13) | 1.9 (30) | 0.0 (0) | 2.0 (18) | 2.5 (13) | 1.3 (12) |
| **Improve work life** | 1.6 (40) | 3.5 (3) | 2.8 (12) | 2.0 (15) | 1.2 (9) | 0.2 (1) | 0.0 (0) | 1.8 (17) | 1.4 (23) | 0.8 (1) | 0.9 (8) | 2.3 (12) | 2.0 (19) |
| **I am planning for my future (e.g., starting a family/finding a partner/planning for retirement)** | 1.4 (36) | 1.2 (1) | 1.6 (7) | 1.9 (14) | 1.1 (8) | 1.4 (6) | 0.0 (0) | 1.4 (13) | 1.4 (23) | 0.8 (1) | 1.8 (16) | 1.3 (7) | 1.3 (12) |
| Future family planning | 52.8 (19) | 100.0 (1) | 85.7 (6) | 78.6 (11) | 12.5 (1) | 0.0 (0) | 0.0 (0) | 53.8 (7) | 52.2 (12) | 0.0 (0) | 56.3 (9) | 42.9 (3) | 58.3 (7) |
| Planning for retirement/old age | 47.2 (17) | 0.0 (0) | 14.3 (1) | 21.4 (3) | 87.5 (7) | 100.0 (6) | 0.0 (0) | 46.2 (6) | 47.8 (11) | 100.0 (1) | 43.8 (7) | 57.1 (4) | 41.7 (5) |
| **Other** | 1.2 (30) | 1.2 (1) | 1.4 (6) | 0.7 (5) | 1.5 (11) | 1.4 (6) | 1.1 (1) | 1.8 (17) | 0.8 (13) | 0.8 (1) | 1.6 (15) | 1.5 (8) | 0.6 (6) |
| **Someone or something inspired me to** | 1.1 (28) | 2.3 (2) | 1.4 (6) | 0.8 (6) | 1.5 (11) | 0.5 (2) | 1.1 (1) | 1.3 (12) | 1.0 (16) | 2.4 (3) | 0.8 (7) | 0.9 (5) | 1.4 (13) |
| Advice or concern | 92.3 (26) | 100.0 (2) | 100.0 (6) | 100.0 (6) | 90.9 (10) | 100.0 (2) | 0.0 (0) | 91.7 (11) | 93.8 (15) | 66.7 (2) | 100.0 (7) | 80.0 (4) | 100.0 (13) |
| Media | 7.1 (2) | 0.0 (0) | 0.0 (0) | 16.7 (1) | 0.0 (0) | 0.0 (0) | 100.0 (1) | 8.3 (1) | 6.3 (1) | 33.3 (1) | 0.0 (0) | 20.0 (1) | 0.0 (0) |
| **I have seen the effects drinking has had on other people** | 0.7 (17) | 2.3 (2) | 0.7 (3) | 0.3 (2) | 1.2 (9) | 0.2 (1) | 0.0 (0) | 0.4 (4) | 0.8 (13) | 0.8 (1) | 0.3 (3) | 0.8 (4) | 0.9 (9) |
| I do not want to end up like someone I know | 100.0 (17) | 100.0 (2) | 100.0 (3) | 100.0 (2) | 100.0 (9) | 100.0 (1) | 0.0 (0) | 100.0 (4) | 100.0 (13) | 100.0 (1) | 100.0 (3) | 100.0 (4) | 100.0 (9) |
| **A significant holiday or event (e.g., birthday or upcoming trip)** | 0.7 (17) | 0.0 (0) | 1.9 (8) | 0.4 (3) | 0.7 (5) | 0.2 (1) | 0.0 (0) | 0.5 (5) | 0.8 (12) | 0.0 (0) | 0.7 (6) | 0.9 (5) | 0.6 (6) |

* Percentage of users for all categories, and for themes within each category may be greater than 100% because each response could be coded multiple times.
