# Supplementary material for: Exploring Motives for Reducing Alcohol Consumption Among Users of an Alcohol Reduction App: Content Analysis
Source: JMIR Public Health Surveill. 2026 Apr 29;12:e88992. doi: 10.2196/88992 (PMC13128053; doi:10.2196/88992)
Supplement: Multimedia Appendix 2 [file publichealth-v12-e88992-s002.docx]

**Frequency of categories, themes, and subthemes reported by Drink Less app users (n=2520)**

| **Category** - Theme - *Subtheme* | % of users (n) | Description |
| --- | --- | --- |
| **I want to improve my physical health** | 52.7% (1329) | See (sub)themes |
| I want to live a healthier lifestyle | 85.0% (1129) | See subthemes |
| *I want to take care of my health* | 98.6% (1113) | Unspecified goal to do so |
| *I want to achieve a health goal (e.g. lower my blood pressure or cholesterol)* | 2.0% (23) | “ |
| I am worried about future health problems | 13.6% (180) | E.g. cancer |
| I have other health problems | 5.4% (72) | See subthemes |
| *It is causing or exacerbating health conditions* | 95.8% (69) | “ |
| *To aid recovery of something* | 4.2% (3) | “ |
| *I am on other medication that I cannot drink with* | 2.8% (2) | “ |
| **I want to feel better in my body** | 32.7% (823) | See (sub)themes |
| Help with weight loss | 87.2% (718) | “ |
| Improve my fitness | 13.7% (113) | “ |
| *Improve my fitness* | 83.2% (94) | In here twice as these are people mentioning wanting to improve fitness in an unspecified way... |
| *Improve at sports/exercise* | 21.2% (24) | ... and these people mention specific sports related goals (e.g. running faster, going to the gym, lifting x amounts of weights etc.) |
| I want to improve how I look | 5.8% (48) | General expression of wanting to look better |
| I want to improve the features on my body | 3.9% (32) | See subthemes |
| *To improve skin* | 81.3% (26) | E.g. skin is oily or irritated |
| *To improve my face* | 15.7% (5) | E.g. face is puffy or swollen |
| *To improve hair* | 6.3% (2) | E.g. hair does not look as nice/sleek |
| *To improve smell/sweating* | 6.3% (2) | “ |
| *To improve eyes* | 3.1% (1) | Have brighter eyes specifically |
| I want to feel more attractive/sexy | 0.4% (3) | “ |
| **I want to improve my mental wellbeing** | 22.5% (567) | See (sub)themes |
| I want to improve my overall wellbeing | 61.0% (346) | General/unspecified want to improve wellbeing |
| *I want to feel better* | 43.6% (151) | Just that but not in relation to i..e physical health (specifically wanting to feel better mentally/generally) |
| *I want to be happier* | 21.7% (75) | “ |
| *Drinking impacts my mood/mindset* | 19.7% (68) | “ |
| *I want to have a better relationship with myself (confidence, self-esteem, self-respect, self-worth)* | 14.7% (51) | “ |
| *It makes me angry/irritable/aggressive/violent* | 5.5% (19) | “ |
| *Less stress* | 2.6% (9) | “ |
| *I want to be calmer/more relaxed* | 1.7% (6) | “ |
| *More patience/less frustration* | 1.4% (5) | Often mentioned in relation to lashing out i.e. with kids and feeling short-tempered |
| Improve mental health | 34.9% (198) | See (sub)themes |
| *Improve mental health* | 42.4% (84) | Same as above because these are general mentions of wanting to improve mental health that are not specified/distinct to things like the subthemes below |
| *Reduce anxiety* | 29.3% (58) | “ |
| *It makes me feel depressed* | 20.2% (40) | “ |
| *I am ready to find a different way to cope with my problems* | 17.7% (35) | In these people specify their drinking as a coping mechanism they have come to find unhealthy and are now ready to try and find ‘healthier’ alternatives |
| *It makes me feel suicidal* | 0.5% (1) | “ |
| I want to feel clearer headed, more alert, focused/be present | 15.3% (87) | “ |
| **I want to regain agency** | 19.2% (483) | See (sub)themes |
| I want to be in control of my drinking | 68.3% (330) | See subthemes |
| *I am worried about how much I drink/becoming/being dependent* | 82.7% (273) | Fear of dependency but also of being dependent already and not realising |
| *I feel guilty/ashamed/disappointed* | 8.2% (27) | .. of my drinking |
| *I usually drink more than I intend (I.e. not being able to say no or stop once I start)* | 5.5% (18) | “ |
| *I have to/I should/need to* | 3.6% (12) | For unspecified reasons |
| *I want to be proud of myself/make others proud* | 2.7% (9) | By controlling my drinking |
| *I want to prove I can do it* | 1.2% (4) | “ |
| I want to be in control (of my actions, life...) | 23.6% (114) | See subthemes |
| *I want to be in control (of my actions, life...)* | 71.1% (81) | Different to above (control of drinking directly) as this specifies wanting control over one's general life and behaviour rather than drinking specifically. Is its own subtheme to theme as others are more specifically tied to i.e. making choices whereas this one is more general want for control not directly about drinking |
| *Alcohol prevents me from making good/better choices* | 28.1% (32) | Or I feel like I make bad/not well-informed choices when I drink |
| *I want to be more responsible/reliable* | 7.9% (9) | I.e. I am not when I drink |
| I do not want drinking to define me | 19.3% (93) | Matter of identity (see subthemes) |
| *I feel more like myself without alcohol/I want to be a different person* | 66.7% (62) | “ |
| *I do not want to embarrass myself or others* | 31.2% (29) | “ |
| *I do not want others to worry/think there is a problem* | 6.5% (6) | “ |
| **I want to live a different life** | 12.7% (321) | See (sub)themes |
| I want to make space in my life for other things | 42.4% (136) | This specifies i.e. spending more time with kids or wanting to do more of a hobby |
| I want to live (longer) | 28.3% (91) | This specifies the wish for a longer life (but expressed positively, not being afraid of death but rather wanting to cherish and prolong life) |
| I want to be more ... | 25.9% (83) | See subthemes |
| *... productive* | 78.3% (65) |  |
| *... successful* | 10.8% (9) |  |
| *... resilient* | 10.8% (9) |  |
| *... creative* | 3.6% (3) |  |
| I want to improve my quality of life | 14.3% (46) | This relates to all expressions of a different life that do not relate to wanting to be different, live longer, or do other things but more a general wish for a ‘better’ life (sometimes directly expressed as ‘better life quality’) |
| **To have better relationships with the people in my life** | 12.3% (309) | See (sub)themes |
| I am doing it for someone | 84.5% (261) | See subthemes |
| *For family* | 79.3% (207) | “ |
| *For friends/unspecified* | 16.5% (43) | (unspecified: included a lot of names so unclear as to who) |
| *For my partner* | 16.5% (43) | “ |
| Drinking affects my relationships/social life | 15.5% (48) | See subthemes |
| *Drinking affects my relationships/social life* | 97.9% (47) | This is its own theme as the second theme felt distinct to this. This one was again more a general expression of social life or relationships being affected but not expressing how |
| *I want others to like me* | 2.1% (1) | “ |
| My drinking affects the way I treat and impact people | 6.8% (21) | See subthemes |
| *My drinking affects the way I treat and impact people* | 81.0% (17) | Double to theme as this is distinct from the second subtheme and more a general expression of feeling like I treat people differently drinking |
| *I have let people down* | 19.0% (4) | “ |
| I want to improve my sex life | 1.3% (4) | “ |
| **A decision that drinking was too expensive** | 9.1% (228) | “ |
| **Restore my energy** | 8.4% (212) | See (sub)themes |
| *Increase my energy/motivation* | 58.0% (123) | “ |
| *I want to sleep better* | 51.4% (109) | “ |
| **I do not want to (re)experience the side effects/consequences** | 6.4% (162) | See (sub)themes |
| *Avoid hangovers/the bad day after* | 47.5% (77) | “ |
| *Avoid memory loss* | 25.9% (42) | “ |
| *Avoid regret* | 13.0% (21) | “ |
| *It makes me ill* | 8.0% (13) | Not related to physical health as these are expressions of consequence that want to be avoided and are not usually related to overall physical health |
| *It ages me* | 6.2% (10) | “ |
| *Avoid black outs/passing out/falling asleep* | 5.6% (9) | “ |
| *Avoid falling over* | 1.2% (2) | “ |
| *I do not want to (re)experience the side effects/consequences* | 1.2% (2) | Distinct and double to theme as these are general expressions of this |
| **Drinking hurts me** | 3.7% (94) | See (sub)themes |
| *It is not good for me* | 36.2% (34) | “ |
| *I am hurting/killing myself* | 25.5% (24) | “ |
| *Drinking is harmful to me* | 16.0% (15) | “ |
| *Ruining life* | 13.8% (13) | “ |
| *Drinking is dangerous/unsafe* | 12.8% (12) | “ |
| **I want to have a better relationship with alcohol** | 2.5% (64) | See (sub)themes |
| I want to moderate my drinking, not abstain | 95.3% (61) | See subthemes |
| *I want to moderate my drinking, not abstain* | 27.9% (17) | Double as these are general expressions of wanting to do so. Not specified like other subthemes |
| *I want to be able to enjoy drinking every once in a while* | 27.9% (17) | “ |
| *I only want to drink a certain amount of units/drinks* | 27.9% (17) | “ |
| *I only want to drink on certain days of the week/implement alcohol free days* | 19.7% (12) | “ |
| *I only want to drink socially, not by myself or at home* | 8.2% (5) | “ |
| *Only on special occasions* | 4.9% (3) | “ |
| *I do not want to drink more than others/I want to be a "normal" drinker* | 3.3% (2) | “ |
| Temporary abstinence (e.g. Dry January) | 6.3% (4) | “ |
| **I have changed my attitude about drinking** | 1.7% (43) | See (sub)themes |
| *Do not enjoy drinking (anymore)* | 44.2% (19) | “ |
| *Drinking does not benefit/serve me anymore* | 37.2% (16) | “ |
| *I am getting older/too old* | 11.6% (5) | “ |
| *It is boring me* | 7.0% (3) | “ |
| *Drinking is uncool* | 2.3% (1) | “ |
| **To support or encourage people in my life to drink less/have a better relationship with alcohol** | 1.7% (43) | See (sub)themes |
| *I want to set a good example* | 88.4% (38) | “ |
| *I want to help/encourage someone else to drink less* | 14.0% (6) | “ |
| **Improve work life** | 1.6% (40) | See (sub)themes |
| *Drinking affects my performance at work* | 92.5% (37) | “ |
| *I want to get a job* | 7.5% (3) | “ |
| **I am planning for my future (e.g., starting a family/finding a partner/planning for retirement)** | 1.4% (36) | See (sub)themes |
| Future family planning | 52.8% (19) | See subthemes |
| *Improve chance to conceive/I am pregnant* | 84.2% (16) | “ |
| *Improve for future partner* | 15.8% (3) | “ |
| Planning for retirement/old age | 47.2% (17) | “ |
| **Other** | 1.2% (30) | See (sub)themes |
| *Because I can or want to* | 43.3% (13) | “ |
| *Quitting something else as well (like smoking)* | 23.3% (7) | “ |
| *I reduced/abstained before* | 16.7% (5) | “ |
| *Experimenting (seeing how it feels)* | 10.0% (3) | “ |
| *Lockdown* | 6.7% (2) | “ |
| *Drinking no longer has the desired effect* | 3.3% (1) | “ |
| *I want to feel able to be honest about my drinking* | 3.3% (1) | “ |
| *I do not want to be faced with legal repercussions* | 3.3% (1) | “ |
| **Someone or something inspired me to** | 1.1% (28) | See (sub)themes |
| Advice or concern | 92.9% (26) | See subthemes |
| *The people in my life are worried about me* | 84.6% (22) | This worry directly inspired reduction attempt |
| *Advice from a doctor/health professional* | 15.4% (4) | “ |
| Media | 7.1% (2) | See subthemes |
| *A radio program* | 50.0% (1) | “ |
| *The Drink Less App* | 50.0% (1) | “ |
| **I have seen the effects drinking has had on other people** | 0.7% (17) | See (sub)themes |
| I do not want to end up like someone I know | 100.0% (17) | See subthemes |
| *I do not want to end up like someone I know* | 58.8% (10) | Double as this is a general expression of this whereas other two subthemes are more distinct expressions of this theme |
| *Someone I know was hurt/died as a result of drinking* | 41.2% (7) | “ |
| *I want to break a cycle of drug abuse that runs in my family* | 17.6% (3) | “ |
| **A significant holiday or event** | 0.7% (17) | “ |
| * Percentage of users for all categories, and for themes within each category may be greater than 100% because each response could be coded multiple times | | |
